# Correlation Between Body Mass Index and Depression/Depression-Like Symptoms Among Different Genders and Races

**DOI:** 10.7759/cureus.21841

**Published:** 2022-02-02

**Authors:** Nathan Badillo, Mohammed Khatib, Payal Kahar, Deepesh Khanna

**Affiliations:** 1 Dr. Kiran C. Patel College of Osteopathic Medicine, Nova Southeastern University, Clearwater, USA; 2 Department of Health Sciences, Florida Gulf Coast University, Fort Myers, USA; 3 Foundational Sciences, Nova Southeastern University Dr. Kiran C. Patel College of Osteopathic Medicine, Clearwater, USA

**Keywords:** body mass index (bmi), race inequities, management of depression in chronic illness, suicidal thoughts, sleep problems, psychiatry and mental health, race-based medicine, gender-based differences, weight loss and obesity, depression

## Abstract

Background

While being overweight is a risk factor for hyperlipidemia, type 2 diabetes, cancer, and heart disease, it can also be a risk factor for depression and vice versa. In this study, we aimed to evaluate the relationship between body mass index (BMI) and the prevalence of depression symptoms between genders and races.

Methodology

A nationally representative sample was utilized to explore the relationship between depression-related symptoms and BMI status by comparing different genders and racial identities. The National Health and Nutrition Examination Survey (NHANES) data, managed by the Centers for Disease Control and Prevention, was used in this study. Data from 2013 to 2016 were included in the analysis. The Patient Health Questionnaire was used to collect information regarding responses to eight primary questions based on gender, race, and BMI status. Statistical analysis was conducted using descriptive analysis and the chi-square test.

Results

Data were presented as percentages. A majority of both men and women who admitted to having depression or depression-like symptoms more than half the days or nearly every day were overweight or obese. However, men had a higher prevalence compared to women for most questions. Statistical analysis showed that among men and women who felt down, depressed, or hopeless nearly every day, 61.5% (χ^2^ = 5.045, p = 0.992) and 50.9% (χ^2^ = 17.186, p = 0.308) were overweight, respectively. Among the races, those who felt down, depressed, or hopeless nearly every day, non-Hispanic Asian individuals had the lowest percentage of being overweight at 47.7% (χ^2^ = 7.099, p = 0.955), while Hispanic individuals other than Mexican Americans had the highest percentage of being overweight at 67.4% (χ^2^ = 8.792, p = 0.721).

Conclusions

Being overweight or obese appears to have a positive relationship with depression and depression-like symptoms for each gender and race. Similarly, individuals who report having depression-like symptoms are likely to be overweight or obese. Further research is needed to determine other differences in etiologies between genders and races, along with determining whether more individuals become depressed due to being overweight or obese or whether more individuals become overweight or obese due to being depressed. The results of this study are limited to the data obtained through NHANES.

## Introduction

In the United States, the incidence and prevalence of depression and other mental illnesses continue to rise. According to a study, 19% of adults experienced a mental illness between 2017 and 2018, with an increase of 1.5 million cases compared to the cases reported between 2016 and 2017 [1]. In addition, suicidal ideation among adults increased by 0.15% during the same period [1]. The pathophysiology of depression can result from a combination of genetic and/or biological factors. Biological factors such as obesity and the associated metabolic disorders can cause depression [2,3]. In addition, environmental factors can contribute to the onset and progression of depression, including chronic stress, acute life events, childhood trauma, and social stressors.

Women have a higher prevalence of developing depression compared to men [4]. Parker and Brotchie reported some possible reasons for the predominance of depressive symptoms in women [5]. According to the authors, women may be more likely to voluntarily reveal their symptoms in the context of available help-seeking components. Meanwhile, the researchers noted that men were more likely to forget or “get past” episodes that could impact their mood, while women were more likely to remember them. Another study reported that several factors can contribute to depression onset and progression and that women are more likely to present with depression than men [6]. Moreover, women are more likely to experience moderate to severe depressive symptoms, present with more depression subtypes, and report more somatic and cognitive-affective symptoms compared to men [6].

Understanding the prevalence of depressive symptoms among men and women of different ethnicities/races remains understudied in the field of mental illness. Therefore, using the Patient Health Questionnaire-9 (PHQ-9), our research group aimed to understand how depression affects populations, as well as how it can be most effectively treated based on gender and race/ethnicity. According to Dr. Karen Martinez, culture plays a role in the expression of thoughts behaviors, and emotions. Hence, there are differences based on gender, culture, and race/ethnicity regarding how depression and anxiety are diagnosed and treated [7]. In a study comparing Asian and Hispanic adolescents, it was found that overweight body image is a significant predictor for the development of depression in Asian girls and boys compared to Hispanic girls and boys [8]. A literature review of 19 studies showed that African American men compared to white men face prolonged disability in association with depression, face certain risk factors for depression, as well as show low utilization of mental health services [9].

There has also been speculation whether minorities are disproportionately impacted by depression and obesity. According to the Centers for Disease Control and Prevention (CDC), in 2021, non-Hispanic black individuals (49.6%) had the highest age-adjusted prevalence of obesity, followed by Hispanic individuals (44.8%), non-Hispanic white individuals (42.2%), and non-Hispanic Asian individuals (17.4%) [10]. Furthermore, racial discrimination may be a factor in the cause and moderation of depressive symptoms, wherein higher levels of depressive symptoms are forecasted by higher perceived prejudice and lower self-reported competence at work [11].

According to Dr. Michael Craig Miller, an assistant professor of psychiatry at Harvard Medical School in Boston, Massachusetts, obesity and depression may be linked because obesity affects parts of the brain that impact mood. It has been shown when individuals are depressed, low energy and motivation may result in decreased activities of daily living and exercise, which can be associated with weight gain [12]. In addition, a study that examined a UK cohort of obese patients and the effect of body mass index (BMI) on depression showed a stepwise pattern of increased risk of depression among individuals with a BMI greater than 30 kg/m^2^, with the risk doubling among patients with a BMI greater than 60 kg/m^2^ [13]. Similar findings were reported by a longitudinal study on adults with a BMI of 40 kg/m^2^ and above. The greater the extreme of one’s BMI, either very high or very low, the higher is the risk of experiencing depressive symptoms, which was more prevalent among women compared to men [14]. This pattern of increased depressive symptoms in those with extreme BMIs was also seen in a study conducted among Korean adults [15]. Because of the correlation between obesity and depression, healthcare professionals should be vigilant about addressing depression regularly, especially with the growing rates of obesity in the United States.

The varying symptomatology associated with depression is more apparent among overweight or obese individuals [16]. Sleep quality and quantity have been demonstrated to be associated with both obesity and depression [16]. Numerous factors can explain why the lack of sleep can lead to weight gain, including increased levels of hormones such as ghrelin and decreased levels of adipose tissue-secreted hormones such as leptin, which alone or in combination can lead to weight gain [16]. Sleep disturbances can negatively impact human organ systems and pathophysiology. A study by Araghi et al. evaluated the associations between sleep quality, mood disorders, and quality of life in extremely obese patients [17]. The study found that over two-thirds of patients with severe obesity reported lackluster sleep quality. The researchers also found associations between poor sleep quality and excessive daytime sleepiness with mood disturbances, while controlling for age, sex, hypertension, diabetes, and obstructive sleep apnea. As such, it is necessary to consider the interactions between multiple depression symptoms, including sleep disturbances and obesity, in the treatment of depression [17].

To further understand the relationship between depression and obesity as it relates to gender and race, in this study, we aimed to explore the relationships between depression-related symptoms and BMI status by comparing men and women as well as racial identities using a nationally representative sample. NHANES survey data from 2013 to 2016 was used. We also utilized the Patient Health Questionnaire (PHQ), in which we accounted for responses to the eight primary questions based on gender and BMI status. Based on previous studies, we anticipated that the highest prevalence of depression symptoms in women would occur at a higher BMI compared to men. In addition, we aimed to explore the impact of BMI status on the prevalence of symptoms among different races. We anticipated that those who are overweight or obese would have more severe depressive symptoms among all races compared to those with normal BMI.

## Materials and methods

The NHANES is a program designed to gauge the health and nutritional status of adults and children in the United States. It includes demographic, socioeconomic, dietary, and health-related questions. The examination component of the NHANES includes medical, dental, and physiological analysis, as well as laboratory tests. The data were collected from 2013 to 2016 for analysis. A total of 20,146 subjects participated within this timeframe. Inclusion criteria for our analysis included being 18 years of age and older. The average height, weight, and BMI were taken into consideration, as well as gender and race.

The nine-item depression instrument PHQ-9 was employed to determine the frequency of depression symptoms over the past two weeks with a follow-up question assessing the overall impairment of the symptoms. Each subject responded with “not at all,” “several days,” “more than half the days,” or “nearly every day” to the following questions: Over the last two weeks, how often have you been bothered by the following problems: Little interest or pleasure in doing things?/Feeling down, depressed, or hopeless?/Trouble falling or staying asleep, or sleeping too much?/Feeling tired or having little energy?/Poor appetite or overeating?/Feeling bad about yourself, or that you are a failure, or have let yourself or your family down?/Trouble concentrating on things, such as reading the newspaper or TV?/Moving or speaking so slowly that other people could have noticed? Or the opposite, being so fidgety or restless that you have been moving around a lot more than usual?/Thoughts that you would be better off dead or hurting yourself in some way?

Subjects’ responses were analyzed according to the following BMI categories: underweight (<18.5 kg/m^2^), normal (18.5-24.9 kg/m^2^), overweight (25-29.9 kg/m^2^), or obese (>30 kg/m^2^).

Statistical analysis

BMI status was compared with individual responses for questions from the PHQ by cross-tabulation (chi-square test), stratified by gender and race. Statistical analysis was performed using SPSS version 26 (IBM Corp., Armonk, NY, USA) [18]. The level of significance was set at 0.05. P-values were reported for all correlations between PHQ items and BMI status for both gender and race.

## Results

Table 1 displays the average age, weight, height, and BMI of the study population according to gender and race. Among the different race categories, non-Hispanic black individuals had the highest mean weight and the highest mean BMI, while non-Hispanic Asian individuals had the lowest mean weight and the lowest mean BMI. Men averaged 11 kg more in weight compared to women, with their BMI being 0.5 kg/m^2^ less than women. The oldest population surveyed in terms of race were non-Hispanic white individuals, while the youngest population surveyed were other race/multiracial individuals.

**Table 1:** Participant characteristics by gender and race. Values are displayed as means ± standard deviation.

	Age (years) (mean)	Weight (kg) (mean)	Height (in) (mean)	BMI (kg/m^2^) (mean)
Gender				
Men	31.2 (±24.7)	85.7 (±20.2)	69.1 (±3.3)	27.8 (±5.9)
Women	32.2 (±24.5)	74.0 (±20.2)	63.7 (±3)	28.3 (±7.3)
Race				
Mexican Americans	27.3 (±23.1)	79.3 (±19.7)	65.1 (±3.9)	29.1 (±6.6)
Other Hispanics	31.1 (±24.1)	77.2 (±18.5)	65.1 (±3.9)	28.3 (±6.1)
Non-Hispanic whites	36.2 (±26.2)	81.5 (±21.4)	67.1 (±4.1)	28 (±6.6)
Non-Hispanic blacks	30.2 (±24)	85.1 (±21.9)	67.1 (±4)	29.3 (±7.1)
Non-Hispanic Asians	33.1 (±22.2)	65.5 (±13.5)	64.8 (±3.8)	24.1 (±4.1)
Other races including multiracial	21.8 (±21.1)	83.8 (±24.9)	67.4 (±4.1)	28.5 (±7.7)

Table 2 shows the correlation between the different BMI categories based on the self-reported height and weight with individuals having trouble falling asleep, staying asleep, or sleeping too much over the last two weeks for each gender and race. Most individuals who had trouble sleeping or were sleeping too much for more than half the days were overweight or obese for both men and women, Mexican Americans, and non-Hispanic blacks. People who had trouble sleeping or were sleeping too much nearly every day were in the normal-weight or overweight category of BMI for men, women, non-Hispanic whites, and Hispanics other than Mexican Americans. Among the obese category, more women than men and more Mexican Americans than any other race had trouble sleeping or were sleeping too much nearly every day. Non-Hispanic Asian individuals had the least percentage under the underweight and obese categories who had trouble sleeping or were sleeping too much nearly every day. Other races, including multiracial, had statistically significant variability among all races.

**Table 2:** Trouble sleeping or sleeping too much versus BMI status. Cell percentages represent the percentage of each BMI category’s response to the question “Over the last two weeks, how often have you been bothered by the following problems: trouble falling or staying asleep, or sleeping too much?.” (i.e., of the men who responded to the question with “several days,” 19.2% had an about right BMI.) Gender: χ^2^ = 20.257, p = 0.162; race: χ^2^ = 20.257, p = 0.162. BMI: body mass index

BMI status					
	Trouble sleeping or sleeping too much	Underweight	About right	Overweight	Obese
		(BMI <18.5)	(BMI 18.5–24.9)	(BMI 25–29.9)	(BMI >30)
Gender					
Men					
	Not at all	27 (0.8%)	655 (20.1%)	1,986 (60.8%)	598 (18.3%)
	Several days	6 (0.5%)	215 (19.2%)	716 (64%)	182 (16.3%)
	More than half the days	8 (2.4%)	63 (19%)	195 (58.9%)	65 (19.6%)
	Nearly every day	5 (1.1%)	87 (18.9%)	298 (64.8%)	70 (15.2%)
Women					
	Not at all	56 (1.7%)	777 (23.3%)	1,782 (53.4%)	722 (21.6%)
	Several days	25 (2.1%)	291 (24.3%)	623 (52%)	260 (21.7%)
	More than half the days	9 (2.4%)	78 (20.9%)	204 (54.7%)	82 (22%)
	Nearly every day	10 (2.2%)	108 (23.5%)	248 (54%)	93 (20.3%)
Race					
Mexican Americans					
	Not at all	9 (0.8%)	171 (15%)	724 (63.4%)	238 (20.8%)
	Several days	2 (0.5%)	67 (16.1%)	273 (65.8%)	73 (17.6%)
	More than half the days	0 (0%)	10 (7.9%)	82 (64.6%)	35 (27.6%)
	Nearly every day	1 (0.6%)	28 (15.6%)	110 (61.5%)	40 (22.3%)
Other Hispanics					
	Not at all	5 (0.7%)	139 (19.8%)	426 (60.8%)	131 (18.7%)
	Several days	5 (2%)	45 (18%)	151 (60.4%)	49 (19.6%)
	More than half the days	0 (0%)	15 (21.4%)	47 (67.1%)	8 (11.4%)
	Nearly every day	1 (1%)	21 (20.6%)	70 (68.6%)	10 (9.8%)
Non-Hispanic whites					
	Not at all	24 (1%)	553 (24%)	1,218 (52.8%)	511 (22.2%)
	Several days	14 (1.7%)	200 (24.7%)	422 (52%)	175 (21.6%)
	More than half the days	7 (2.9%)	61 (25.1%)	122 (50.2%)	53 (21.8%)
	Nearly every day	3 (1%)	71 (23.2%)	168 (54.9%)	64 (20.9%)
Non-Hispanic blacks					
	Not at all	20 (1.4%)	239 (16.6%)	842 (58.4%)	341 (23.6%)
	Several days	0 (0%)	75 (15.1%)	307 (61.6%)	116 (23.3%)
	More than half the days	4 (2.5%)	26 (16.5%)	89 (56.3%)	39 (24.7%)
	Nearly every day	3 (1.5%)	31 (15.7%)	125 (63.5%)	38 (19.3%)
Non-Hispanic Asians					
	Not at all	21 (3%)	282 (40.7%)	346 (49.9%)	44 (6.3%)
	Several days	10 (4%)	104 (41.4%)	127 (50.6%)	10 (4%)
	More than half the days	4 (5.3%)	23 (30.3%)	43 (56.6%)	6 (7.9%)
	Nearly every day	4 (4.3%)	34 (36.2%)	50 (53.2%)	6 (6.4%)
Other races including multiracial					
	Not at all	4 (1.3%)	48 (15%)	212 (66.5%)	55 (17.2%)
	Several days	0 (0%)	15 (16.1%)	59 (63.4%)	19 (20.4%)
	More than half the days	2 (6.7%)	6 (20%)	16 (53.3%)	6 (20%)
	Nearly every day	3 (7.3%)	10 (24.4%)	23 (56.1%)	5 (12.2%)

Table 3 shows the correlation between BMI categories based on the self-reported height and weight among individuals who felt tired or had little energy over the prior two weeks for each gender and race. Those who felt tired or had little energy (for more than half the days) were mostly categorized as overweight or about being the right weight among women, Hispanics (other than Mexican Americans), non-Hispanic Asians, and multiracial. Individuals who felt tired or had little energy were mostly categorized as overweight or about the right weight for men, women, non-Hispanic whites, and non-Hispanic Asians. Non-Hispanic Asians had the highest percentage of those who felt tired or had little energy and were categorized as having a normal BMI, while non-Hispanic blacks had the highest percentage of individuals categorized as obese. Non-Hispanic Asians also had the lowest percentage of those who felt tired or had little energy (every day) among those categorized as being obese. Within the obese category, more women compared to men felt tired or had little energy (nearly every day).

**Table 3:** Feeling tired or having little energy versus BMI status. Cell percentages represent the percentage of each BMI category’s response to the question “Over the last two weeks, how often have you been bothered by the following problems: feeling tired or having little energy?.” (i.e., of the men who responded to the question with “several days,” 20.6% had an about right BMI.) Gender: χ^2^ = 24.754, p = 0.016; race: χ^2^ = 24.754, p = 0.016. BMI: body mass index

		BMI status			
	Feeling tired or having little energy	Underweight (BMI <18.5)	About right (BMI 18.5–24.9)	Overweight (BMI 25–29.9)	Obese (BMI >30)
Gender					
Men					
	Not at all	18 (0.7%)	492 (19.7%)	1,529 (61.3%)	457 (18.3%)
	Several days	18 (1%)	364 (20.6%)	1,077 (61%)	306 (17.3%)
	More than half the days	7 (1.5%)	70 (15.4%)	295 (65%)	82 (18.1%)
	Nearly every day	3 (0.7%)	95 (20.6%)	294 (63.8%)	69 (15%)
Women					
	Not at all	44 (1.7%)	593 (22.7%)	1,404 (53.8%)	571 (21.9%)
	Several days	38 (2%)	458 (24.7%)	973 (52.4%)	387 (20.9%)
	More than half the days	11 (2.5%)	90 (20.8%)	245 (56.7%)	86 (19.9%)
	Nearly every day	7 (1.5%)	114 (24.4%)	235 (50.2%)	112 (23.9%)
Race					
Mexican Americans					
	Not at all	7 (0.8%)	125 (14.6%)	548 (63.9%)	178 (20.7%)
	Several days	5 (0.7%)	108 (15.8%)	426 (62.5%)	143 (21%)
	More than half the days	0 (0%)	17 (11.5%)	101 (68.2%)	30 (20.3%)
	Nearly every day	0 (0%)	26 (14.9%)	115 (65.7%)	34 (19.4%)
Other Hispanics					
	Not at all	4 (0.7%)	104 (18.7%)	344 (61.8%)	105 (18.9%)
	Several days	6 (1.6%)	84 (22.6%)	217 (58.3%)	65 (17.5%)
	More than half the days	1 (1%)	18 (18%)	68 (68%)	13 (13%)
	Nearly every day	0 (0%)	15 (15.8%)	65 (68.4%)	15 (15.8%)
Non-Hispanic whites					
	Not at all	21 (1.2%)	416 (23.4%)	947 (53.2%)	397 (22.3%)
	Several days	18 (1.4%)	322 (25.6%)	658 (52.3%)	261 (20.7%)
	More than half the days	5 (1.6%)	58 (18.9%)	171 (55.7%)	73 (23.8%)
	Nearly every day	4 (1.3%)	89 (28%)	153 (48.1%)	72 (22.6%)
Non-Hispanic blacks					
	Not at all	12 (1.1%)	185 (16.4%)	661 (58.7%)	268 (23.8%)
	Several days	5 (0.6%)	136 (17.5%)	459 (58.9%)	179 (23%)
	More than half the days	6 (3.2%)	24 (12.6%)	122 (64.2%)	38 (20%)
	Nearly every day	4 (2%)	26 (13%)	122 (61%)	48 (24%)
Non-Hispanic Asians					
	Not at all	16 (2.9%)	219 (40.1%)	275 (50.4%)	36 (6.6%)
	Several days	17 (4.5%)	147 (39.2%)	192 (51.2%)	19 (5.1%)
	More than half the days	4 (4.3%)	33 (35.1%)	50 (53.2%)	7 (7.4%)
	Nearly every day	2 (2%)	45 (45%)	49 (49%)	4 (4%)
Other races including multiracial					
	Not at all	2 (0.8%)	36 (15%)	158 (65.8%)	44 (18.3%)
	Several days	5 (3.2%)	25 (16.2%)	98 (63.6%)	26 (16.9%)
	More than half the days	2 (4.3%)	10 (21.3%)	28 (59.6%)	7 (14.9%)
	Nearly every day	0 (0%)	8 (19.5%)	25 (61%)	8 (19.5%)

Table 4 shows the correlation between BMI categories based on the self-reported height and weight of individuals who had a poor appetite or overate for the last two weeks for each gender and race. Those who had a poor appetite or overate for more than half the days were either overweight or obese for men, Mexican Americans, non-Hispanic whites, non-Hispanic blacks, and multiracial. Those who had a poor appetite or overate nearly every day were overweight or normal-weight for men, women, Hispanics other than Mexican Americans, non-Hispanic whites, non-Hispanic Asians, and multiracial. Non-Hispanic Asians had the highest percentage of those who had a poor appetite or overate nearly every day were in the normal-weight category, Hispanics other than Mexican Americans who had the highest percentage of those who had a poor appetite or overate were overweight, and non-Hispanic blacks who had the highest percentage of those who had a poor appetite or overate every day were obese. Non-Hispanic Asians had the lowest percentage of those who had a poor appetite or overate every day and were obese. Among the obese category, more women than men had a poor appetite or overate nearly every day.

**Table 4:** Poor appetite or overeating versus BMI status. Cell percentages represent the percentage of each BMI category’s response to the question “Over the last two weeks, how often have you been bothered by the following problems: poor appetite or overeating?” (i.e., of the men who responded to the question with “several days,” 19% had an about right BMI). BMI: body mass index

		BMI status			
	Poor appetite or overeating	Underweight (BMI <18.5)	About right (BMI 18.5–24.9)	Overweight (BMI 25–29.9)	Obese (BMI >30)
Gender					
Men					
	Not at all	35 (0.9%)	767 (20%)	2,344 (61.2%)	684 (17.9%)
	Several days	5 (0.6%)	159 (19%)	537 (64.3%)	134 (16%)
	More than half the days	4 (1.6%)	50 (19.4%)	142 (55%)	62 (24%)
	Nearly every day	2 (0.8%)	44 (17.5%)	171 (68.1%)	34 (13.5%)
Women					
	Not at all	76 (1.9%)	915 (22.8%)	2,134 (53.3%)	881 (22%)
	Several days	18 (2.1%)	215 (24.9%)	451 (52.3%)	179 (20.7%)
	More than half the days	4 (1.5%)	65 (24.7%)	141 (53.6%)	53 (20.2%)
	Nearly every day	2 (0.9%)	59 (25.2%)	129 (55.1%)	44 (18.8%)
Race					
Mexican Americans					
	Not at all	8 (0.6%)	199 (14.5%)	873 (63.7%)	291 (21.2%)
	Several days	4 (1.3%)	54 (17.6%)	195 (63.7%)	53 (17.3%)
	More than half the days	0 (0%)	10 (10.4%)	61 (63.5%)	25 (26%)
	Nearly every day	0 (0%)	13 (14.4%)	60 (66.7%)	17 (18.9%)
Other Hispanics					
	Not at all	8 (1%)	163 (19.8%)	498 (60.5%)	154 (18.7%)
	Several days	2 (1%)	40 (20.9%)	118 (61.8%)	31 (16.2%)
	More than half the days	1 (1.7%)	11 (18.6%)	39 (66.1%)	8 (13.6%)
	Nearly every day	0 (0%)	7 (13.7%)	39 (76.5%)	5 (9.8%)
Non-Hispanic whites					
	Not at all	40 (1.5%)	645 (23.7%)	1,426 (52.4%)	608 (22.4%)
	Several days	4 (0.7%)	148 (25.7%)	307 (53.3%)	117 (20.3%)
	More than half the days	4 (2%)	46 (23.4%)	98 (49.7%)	49 (24.9%)
	Nearly every day	0 (0%)	45 (26.3%)	97 (56.7%)	29 (17%)
Non-Hispanic blacks					
	Not at all	21 (1.2%)	284 (16.5%)	1,018 (59.1%)	400 (23.2%)
	Several days	1 (0.3%)	54 (14.7%)	226 (61.6%)	86 (23.4%)
	More than half the days	2 (1.9%)	22 (20.8%)	57 (53.8%)	25 (23.6%)
	Nearly every day	3 (3.1%)	11 (11.2%)	62 (63.3%)	22 (22.4%)
Non-Hispanic Asians					
	Not at all	29 (3.4%)	335 (39.7%)	427 (50.7%)	52 (6.2%)
	Several days	8 (4.5%)	62 (35.2%)	96 (54.5%)	10 (5.7%)
	More than half the days	1 (2.2%)	23 (51.1%)	20 (44.4%)	1 (2.2%)
	Nearly every day	1 (2%)	23 (46%)	23 (46%)	3 (6%)
Other races including multiracial					
	Not at all	5 (1.4%)	56 (15.7%)	236 (66.1%)	60 (16.8%)
	Several days	4 (4.9%)	16 (19.5%)	46 (56.1%)	16 (19.5%)
	More than half the days	0 (0%)	3 (16.7%)	8 (44.4%)	7 (38.9%)
	Nearly every day	0 (0%)	4 (16%)	19 (76%)	2 (8%)

Table 5 shows the correlation between BMI categories based on the self-reported height and weight among individuals who felt bad about themselves for the past two weeks based on gender and race. Individuals who felt bad about themselves (more than half the days) were categorized as being overweight or about the right weight among men, women, Mexican Americans, Hispanics (other than Mexican Americans), and non-Hispanic Asians. Individuals who felt bad about themselves (nearly every day) were categorized as being overweight for both men and women, as well as among all races. Non-Hispanic Asians had the highest percentage of those who felt bad about themselves (nearly every day) categorized as about being the right weight and the lowest percentage categorized as being obese. Non-Hispanic whites had the highest percentage of those who felt bad about themselves (nearly every day) categorized as being obese. Within the obese category, more women compared to men felt bad about themselves (nearly every day).

**Table 5:** Feeling bad about yourself versus BMI status. Cell percentages represent the percentage of each BMI category’s response to the question “Over the last two weeks, how often have you been bothered by the following problems: feeling bad about yourself, or that you are a failure, or have let yourself or your family down?” (i.e., of the men who responded to the question with “several days,” 20.4% had an about right BMI.) Gender: χ^2^ = 11.578, p = 0.711; race: χ^2^ = 11.578, p = 0.711. BMI: body mass index

		BMI status			
	Feeling bad about yourself	Underweight (BMI <18.5)	About right (BMI 18.5–24.9)	Overweight (BMI 25–29.9)	Obese (BMI >30)
Gender					
Men					
	Not at all	38 (0.9%)	841 (19.7%)	2,628 (61.7%)	753 (17.7%)
	Several days	6 (1%)	119 (20.4%)	352 (60.5%)	105 (18%)
	More than half the days	0 (0%)	31 (18.7%)	106 (63.9%)	29 (17.5%)
	Nearly every day	2 (1.2%)	29 (17.3%)	109 (64.9%)	28 (16.7%)
Women					
	Not at all	80 (1.8%)	1047 (23.3%)	2,390 (53.1%)	980 (21.8%)
	Several days	16 (2.7%)	142 (23.9%)	322 (54.1%)	115 (19.3%)
	More than half the days	1 (0.7%)	31 (23%)	734 (54.1%)	30 (22.2%)
	Nearly every day	3 (2.2%)	32 (23.7%)	68 (50.4%)	32 (23.7%)
Race					
Mexican Americans					
	Not at all	9 (0.6%)	228 (14.8%)	985 (63.9%)	319 (20.7%)
	Several days	2 (0.9%)	30 (13.7%)	141 (64.4%)	46 (21%)
	More than half the days	0 (0%)	12 (23.5%)	30 (58.8%)	9 (17.6%)
	Nearly every day	1 (1.9%)	6 (11.3%)	34 (64.2%)	12 (22.6%)
Other Hispanics					
	Not at all	8 (0.9%)	188 (20%)	574 (61.1%)	170 (18.1%)
	Several days	3 (2.4%)	23 (18.5%)	78 (62.9%)	20 (16.1%)
	More than half the days	0 (0%)	4 (14.8%)	21 (77.8%)	2 (7.4%)
	Nearly every day	0 (0%)	6 (18.2%)	21 (63.6%)	6 (18.2%)
Non-Hispanic whites					
	Not at all	38 (1.3%)	730 (24.1%)	1,596 (52.7%)	666 (22%)
	Several days	8 (1.9%)	110 (25.8%)	222 (52.1%)	86 (20.2%)
	More than half the days	0 (0%)	23 (20.7%)	59 (53.2%)	29 (26.1%)
	Nearly every day	2 (2.1%)	21 (21.9%)	51 (53.1%)	22 (22.9%)
Non-Hispanic blacks					
	Not at all	22 (1.2%)	313 (16.4%)	1,116 (58.6%)	454 (23.8%)
	Several days	3 (1.2%)	41 (16.5%)	154 (61.8%)	51 (20.5%)
	More than half the days	1 (1.6%)	5 (7.8%)	45 (70.3%)	13 (20.3%)
	Nearly every day	1 (1.3%)	11 (14.7%)	47 (62.7%)	16 (21.3%)
Non-Hispanic Asians					
	Not at all	33 (3.6%)	364 (39.2%)	476 (51.3%)	55 (5.9%)
	Several days	5 (4.2%)	49 (41.5%)	57 (48.3%)	7 (5.9%)
	More than half the days	0 (0%)	14 (43.8%)	16 (50%)	2 (6.3%)
	Nearly every day	1 (2.9%)	15 (42.9%)	17 (48.6%)	2 (5.7%)
Other races including multiracial					
	Not at all	8 (1.9%)	65 (15.7%)	271 (65.6%)	69 (16.7%)
	Several days	1 (2.4%)	8 (19.5%)	22 (53.7%)	10 (24.4%)
	More than half the days	0 (0%)	4 (25%)	8 (50%)	4 (25%)
	Nearly every day	0 (0%)	2 (18.2%)	7 (63.6%)	2 (18.2%)

Table 6 shows the correlation between BMI categories based on the self-reported height and weight of individuals who have trouble concentrating on things over the last two weeks for each gender and race. Those who had trouble concentrating on things more than half of the days and were either overweight or obese included men, Mexican Americans, Hispanics other than Mexican Americans, non-Hispanic blacks, and multiracial. Those who had trouble concentrating on things nearly every day and were overweight or about the right weight included men, women, non-Hispanic whites, non-Hispanic Asians, and multiracial. Non-Hispanic Asians had the highest percentage of those who have trouble concentrating every day and were in the right-weight category. Hispanics other than Mexican Americans had the highest percentage of those who had trouble concentrating every day and were overweight. Non-Hispanic whites had the highest percentage of those who had trouble concentrating every day and were obese. Among the obese category, more women than men had trouble concentrating nearly every day.

**Table 6:** Trouble concentrating on things versus BMI status. Cell percentages represent the percentage of each BMI category’s response to the question “Over the last two weeks, how often have you been bothered by the following problems: trouble concentrating on things, such as reading the newspaper or watching TV?.” (i.e., of the men who responded to the question with “several days,” 20.7% had an about right BMI.) Gender: χ^2^ = 12.837, p = 0.615; race: χ^2^ = 12.837, p = 0.615. BMI: body mass index

		BMI status			
	Trouble concentrating on things	Underweight (BMI <18.5)	About right (BMI 18.5–24.9)	Overweight (BMI 25–29.9)	Obese (BMI >30)
Gender					
Men					
	Not at all	40 (0.9%)	856 (19.9%)	2,659 (61.8%)	750 (17.4%)
	Several days	5 (1%)	104 (20.7%)	305 (60.6%)	89 (17.7%)
	More than half the days	1 (0.6%)	30 (16.6%)	104 (57.5%)	46 (25.4%)
	Nearly every day	0 (0%)	30 (16.3%)	125 (67.9%)	29 (15.8%)
Women					
	Not at all	82 (1.9%)	1,024 (23.1%)	2,357 (53.2%)	968 (21.8%)
	Several days	15 (2.5%)	142 (23.5%)	327 (54%)	121 (20%)
	More than half the days	2 (1.3%)	40 (25.2%)	84 (52.8%)	33 (20.8%)
	Nearly every day	1 (0.6%)	49 (28.7%)	86 (50.3%)	35 (20.5%)
Race					
Mexican Americans					
	Not at all	11 (0.7%)	223 (14.5%)	986 (63.9%)	323 (20.9%)
	Several days	0 (0%)	37 (18.6%)	126 (63.3%)	36 (18.1%)
	More than half the days	1 (1.9%)	6 (11.1%)	34 (63%)	13 (24.1%)
	Nearly every day	0 (0%)	10 (15.4%)	42 (64.6%)	13 (20%)
Other Hispanics					
	Not at all	9 (1%)	195 (20.9%)	562 (60.3%)	166 (17.8%)
	Several days	1 (0.8%)	19 (16.1%)	75 (63.6%)	23 (19.5%)
	More than half the days	1 (2.6%)	3 (7.9%)	29 (76.3%)	5 (13.2%)
	Nearly every day	0 (0%)	4 (11.1%)	28 (77.8%)	4 (11.1%)
Non-Hispanic whites					
	Not at all	40 (1.3%)	712 (23.6%)	1,611 (53.3%)	659 (21.8%)
	Several days	6 (1.6%)	103 (27%)	192 (50.4%)	80 (21%)
	More than half the days	1 (0.7%)	38 (26.6%)	66 (46.2%)	38 (26.6%)
	Nearly every day	1 (0.8%)	32 (27.1%)	59 (50%)	26 (22%)
Non-Hispanic blacks					
	Not at all	23 (1.2%)	314 (16.4%)	1,137 (59.3%)	444 (23.1%)
	Several days	4 (1.8%)	32 (14.2%)	134 (59.3%)	56 (24.8%)
	More than half the days	0 (0%)	9 (13.4%)	39 (58.2%)	19 (28.4%)
	Nearly every day	0 (0%)	15 (18.3%)	52 (63.4%)	15 (18.3%)
Non-Hispanic Asians					
	Not at all	33 (3.6%)	371 (40.4%)	461 (50.2%)	54 (5.9%)
	Several days	6 (4.7%)	46 (35.7%)	72 (55.8%)	5 (3.9%)
	More than half the days	0 (0%)	13 (52%)	10 (40%)	2 (8%)
	Nearly every day	0 (0%)	14 (33.3%)	23 (54.8%)	5 (11.9%)
Other races including multiracial					
	Not at all	6 (1.5%)	65 (16.2%)	259 (64.4%)	72 (17.9%)
	Several days	3 (5.5%)	9 (16.4%)	33 (60%)	10 (18.2%)
	More than half the days	0 (0%)	1 (7.7%)	10 (76.9%)	2 (15.4%)
	Nearly every day	0 (0%)	4 (33.3%)	7 (58.3%)	1 (8.3%)

Table 7 shows the correlation between BMI categories based on the self-reported height and weight of individuals who moved or spoke slowly or too fast over the last two weeks for each gender and race. Those who moved slowly or spoke slowly or too fast more than half the days were overweight for each gender and race. Those who moved slowly or spoke slowly or too fast nearly every day and were overweight or about the right weight included men, women, Mexican Americans, non-Hispanic whites, and non-Hispanic Asians. Non-Hispanic Asians had the highest percentage of those who moved or spoke slowly or too fast nearly every day and were in the right-weight category. Non-Hispanic whites had the highest percentage of those who moved or spoke slowly or too fast nearly every day and were overweight. Hispanics other than Mexican Americans had the highest percentage of those who moved or spoke slowly or too fast nearly every day and were overweight/obese. Among the obese category, more women than men moved or spoke slowly or too fast nearly every day. Hispanics other than Mexican Americans and non-Hispanic whites had statistically significant variability among all races.

**Table 7:** Moving or speaking slowly or too fast versus BMI status. Cell percentages represent the percentage of each BMI category’s response to the question “Over the last two weeks, how often have you been bothered by the following problems: moving or speaking so slowly that other people could have noticed? Or the opposite, being so fidgety or restless that you have been moving around a lot more than usual?.” (i.e., of the men who responded to the question with “several days,” 24.1% had an about right BMI.) Gender: χ^2^ = 20.049, p = 0.170; race: χ^2^ = 20.049, p = 0.170. BMI: body mass index

		BMI status			
	Moving or speaking slowly or too fast	Underweight (BMI <18.5)	About right (BMI 18.5–24.9)	Overweight (BMI 25–29.9)	Obese (BMI >30)
Gender					
Men					
	Not at all	40 (0.9%)	893 (19.5%)	2,821 (61.6%)	829 (18.1%)
	Several days	4 (1.1%)	90 (24.1%)	227 (60.7%)	53 (14.2%)
	More than half the days	2 (1.6%)	20 (16.4%)	78 (63.9%)	22 (18%)
	Nearly every day	0 (0%)	17 (17.9%)	69 (72.6%)	9 (9.5%)
Women					
	Not at all	91 (1.9%)	1,127 (23.5%)	2,538 (52.8%)	1048 (21.8%)
	Several days	7 (2.1%)	79 (23.2%)	191 (56%)	64 (18.8%)
	More than half the days	0 (0%)	23 (20.5%)	63 (56.3%)	26 (23.2%)
	Nearly every day	2 (1.9%)	24 (22.4%)	63 (58.9%)	18 (16.8%)
Race					
Mexican Americans					
	Not at all	12 (0.7%)	243 (14.6%)	1,061 (63.7%)	349 (21%)
	Several days	0 (0%)	17 (14.3%)	80 (67.2%)	22 (18.5%)
	More than half the days	0 (0%)	7 (19.4%)	21 (58.3%)	8 (22.2%)
	Nearly every day	0 (0%)	9 (21.4%)	27 (64.3%)	6 (14.3%)
Other Hispanics					
	Not at all	10 (1%)	203 (20.2%)	602 (60%)	188 (18.7%)
	Several days	0 (0%)	11 (13.8%)	65 (81.3%)	4 (5%)
	More than half the days	1 (4.3%)	4 (17.4%)	16 (69.6%)	2 (8.7%)
	Nearly every day	0 (0%)	3 (17.6%)	11 (64.7%)	3 (17.6%)
Non-Hispanic whites					
	Not at all	41 (1.3%)	782 (24%)	1,716 (52.7%)	717 (22%)
	Several days	7 (2.8%)	73 (29.2%)	119 (47.6%)	51 (20.4%)
	More than half the days	0 (0%)	16 (18%)	47 (52.8%)	26 (29.2%)
	Nearly every day	0 (0%)	13 (18.8%)	47 (68.1%)	9 (13%)
Non-Hispanic blacks					
	Not at all	25 (1.2%)	334 (16.3%)	1,197 (58.5%)	491 (24%)
	Several days	1 (0.7%)	24 (15.7%)	98 (64.1%)	30 (19.6%)
	More than half the days	1 (1.9%)	6 (11.5%)	40 (76.9%)	5 (9.6%)
	Nearly every day	0 (0%)	6 (14.6%)	28 (68.3%)	7 (17.1%)
Non-Hispanic Asians					
	Not at all	36 (3.7%)	393 (39.9%)	501 (50.9%)	55 (5.6%)
	Several days	2 (2.6%)	33 (42.9%)	37 (48.1%)	5 (6.5%)
	More than half the days	0 (0%)	9 (32.1%)	13 (46.4%)	6 (21.4%)
	Nearly every day	1 (4.2%)	8 (33.3%)	15 (62.5%)	0 (0%)
Other races including multiracial					
	Not at all	7 (1.6%)	65 (15.1%)	282 (65.4%)	77 (17.9%)
	Several days	1 (2.8%)	11 (30.6%)	19 (52.8%)	5 (13.9%)
	More than half the days	0 (0%)	1 (16.7%)	4 (66.7%)	1 (16.7%)
	Nearly every day	1 (11.1%)	2 (22.2%)	4 (44.4%)	2 (22.2%)

Table 8 shows the correlation between BMI categories based on the self-reported height and weight of individuals who had thoughts that they would be better off dead over the last two weeks for each gender and race. Those who had thoughts that they would be better off dead more than half the days were in the overweight category for each gender and race. Those who had thoughts that they would be better off dead nearly every day were also in the overweight category for each gender and race. Non-Hispanic Asians had the highest percentage of those who had thoughts that they would be better off dead nearly every day and were in the right-weight category. Mexican Americans had the highest percentage of those who had thoughts that they would be better off dead nearly every day and were overweight. Hispanics other than Mexican Americans had the highest percentage of those who had thoughts that they would be better off dead nearly every day and were obese. Among the obese category, more women than men had thoughts that they would be better off dead nearly every day.

**Table 8:** Thought you would be better off dead versus BMI status. Cell percentages represent the percentage of each BMI category’s response to the question “Over the last two weeks, how often have you been bothered by the following problems: thoughts that you would be better off dead or of hurting yourself in some way?” (i.e., of the men who responded to the question with “several days,” 20.7% had an about right BMI. Gender: χ^2^ = 7.011, p = 0.957; race: χ^2^ = 7.011, p = 0.957. BMI: body mass index

		BMI status			
	Thought you would be better off dead	Underweight (BMI <18.5)	About right (BMI 18.5–24.9)	Overweight (BMI 25–29.9)	Obese (BMI >30)
Gender					
Men					
	Not at all	44 (0.9%)	980 (19.7%)	3,066 (61.7%)	880 (17.7%)
	Several days	1 (0.7%)	28 (20.7%)	85 (63%)	21 (15.6%)
	More than half the days	1 (2.6%)	9 (23.1%)	23 (59%)	6 (15.4%)
	Nearly every day	0 (0%)	3 (10.7%)	19 (67.9%)	6 (21.4%)
Women					
	Not at all	95 (1.8%)	1208 (23.3%)	2,768 (53.4%)	1,117 (21.5%)
	Several days	4 (3.5%)	28 (24.8%)	60 (53.1%)	21 (18.6%)
	More than half the days	0 (0%)	6 (20.7%)	14 (48.3%)	9 (31%)
	Nearly every day	1 (2.9%)	10 (29.4%)	14 (41.2%)	9 (26.5%)
Race					
Mexican Americans					
	Not at all	11 (0.6%)	265 (14.7%)	1,153 (64.1%)	371 (20.6%)
	Several days	1 (2.3%)	7 (15.9%)	28 (63.6%)	8 (18.2%)
	More than half the days	0 (0%)	2 (18.2%)	4 (36.4%)	5 (45.5%)
	Nearly every day	0 (0%)	1 (14.3%)	5 (71.4%)	1 (14.3%)
Other Hispanics					
	Not at all	10 (0.9%)	209 (19.4%)	663 (61.7%)	193 (18%)
	Several days	1 (3.6%)	9 (32.1%)	16 (57.1%)	2 (7.1%)
	More than half the days	0 (0%)	3 (30%)	7 (70%)	0 (0%)
	Nearly every day	0 (0%)	0 (0%)	7 (70%)	3 (30%)
Non-Hispanic whites					
	Not at all	45 (1.3%)	853 (24.2%)	1,858 (52.7%)	769 (21.8%)
	Several days	2 (2.2%)	21 (22.8%)	48 (52.2%)	21 (22.8%)
	More than half the days	0 (0%)	3 (13.6%)	13 (59.1%)	6 (27.3%)
	Nearly every day	1 (4.5%)	6 (27.3%)	10 (45.5%)	5 (22.7%)
Non-Hispanic blacks					
	Not at all	27 (1.2%)	359 (16.1%)	1,319 (59.3%)	519 (23.3%)
	Several days	0 (0%)	3 (7.3%)	31 (75.6%)	7 (17.1%)
	More than half the days	0 (0%)	5 (35.7%)	5 (35.7%)	4 (28.6%)
	Nearly every day	0 (0%)	3 (20%)	8 (53.3%)	4 (26.7%)
Non-Hispanic Asians					
	Not at all	37 (3.5%)	425 (39.9%)	539 (50.7%)	63 (5.9%)
	Several days	1 (2.9%)	14 (41.2%)	17 (50%)	2 (5.9%)
	More than half the days	1 (11.1%)	2 (22.2%)	6 (66.7%)	0 (0%)
	Nearly every day	0 (0%)	3 (42.9%)	3 (42.9%)	1 (14.3%)
Other races including multiracial					
	Not at all	9 (1.9%)	77 (16.4%)	302 (64.3%)	82 (17.4%)
	Several days	0 (0%)	2 (22.2%)	5 (55.6%)	2 (22.2%)
	More than half the days	0 (0%)	0 (0%)	2 (100%)	0 (0%)
	Nearly every day	0 (0%)	0 (0%)	0 (0%)	1 (100%)

Table 9 shows the correlation between BMI categories based on the self-reported height and weight of individuals who felt down, depressed, or hopeless over the last two weeks for each gender and race. Those who felt down, depressed, or hopeless more than half the days were in the overweight or right-weight categories for men, women, Hispanics other than Mexican Americans, non-Hispanic blacks, non-Hispanic Asians, and multiracial. Those who felt down, depressed, or hopeless nearly every day were in the overweight category for all genders and races. Non-Hispanic Asians had the highest percentage of those who felt down, depressed, or hopeless nearly every day fall and were in the right-weight category. Hispanics other than Mexican Americans had the highest percentage of those who felt down, depressed, or hopeless nearly every day and were overweight. Mexican Americans had the highest percentage of those who felt down, depressed, or hopeless nearly every day and were obese.

**Table 9:** Feeling down, depressed, or hopeless versus BMI status. Cell percentages represent the percentage of each BMI category’s response to the question “Over the last two weeks, how often have you been bothered by the following problems: feeling down, depressed, or hopeless?.” (i.e., of the men who responded to the question with “several days,” 18.9% had an about right BMI.) Gender: χ^2^ = 11.651, p = 0.705; race: χ^2^ = 11.651, p = 0.705. BMI: body mass index

		BMI status			
	Feeling down, depressed, or hopeless	Underweight (BMI <18.5)	About right (BMI 18.5–24.9)	Overweight (BMI 25–29.9)	Obese (BMI >30)
Gender					
Men					
	Not at all	33 (0.8%)	772 (19.8%)	2,398 (61.5%)	696 (17.9%)
	Several days	10 (1.2%)	164 (18.9%)	540 (62.3%)	153 (17.6%)
	More than half the days	2 (0.9%)	44 (19.8%)	141 (63.5%)	35 (15.8%)
	Nearly every day	1 (0.5%)	41 (21.9%)	115 (61.5%)	30 (16%)
Women					
	Not at all	76 (1.9%)	933 (22.9%)	2,168 (53.1%)	905 (22.2%)
	Several days	15 (1.7%)	235 (25.9%)	476 (52.4%)	182 (20%)
	More than half the days	5 (2.5%)	45 (22.3%)	123 (60.9%)	29 (14.4%)
	Nearly every day	4 (2.3%)	40 (23.1%)	88 (50.9%)	41 (23.7%)
Race					
Mexican Americans					
	Not at all	10 (0.7%)	211 (14.9%)	899 (63.3%)	300 (21.1%)
	Several days	2 (0.6%)	47 (15%)	202 (64.5%)	62 (19.8%)
	More than half the days	0 (0%)	7 (9.3%)	58 (77.3%)	10 (13.3%)
	Nearly every day	0 (0%)	11 (20.4%)	29 (53.7%)	14 (25.9%)
Other Hispanics					
	Not at all	8 (0.9%)	176 (20.7%)	509 (60%)	156 (18.4%)
	Several days	1 (0.5%)	32 (17.6%)	119 (65.4%)	30 (16.5%)
	More than half the days	1 (2%)	7 (14.3%)	36 (73.5%)	5 (10.2%)
	Nearly every day	1 (2.3%)	6 (14%)	29 (67.4%)	7 (16.3%)
Non-Hispanic whites					
	Not at all	37 (1.3%)	655 (23.8%)	1,440 (52.4%)	617 (22.4%)
	Several days	7 (1.1%)	169 (25.8%)	354 (54%)	125 (19.1%)
	More than half the days	1 (0.7%)	33 (22.8%)	78 (53.8%)	33 (22.8%)
	Nearly every day	3 (2.6%)	28 (24.3%)	57 (49.6%)	27 (23.5%)
Non-Hispanic blacks					
	Not at all	20 (1.1%)	277 (15.8%)	1,049 (59.8%)	408 (23.3%)
	Several days	3 (0.8%)	67 (18.4%)	200 (54.8%)	95 (26%)
	More than half the days	3 (3.4%)	15 (16.9%)	57 (64%)	14 (15.7%)
	Nearly every day	1 (1.1%)	12 (13.6%)	58 (65.9%)	17 (19.3%)
Non-Hispanic Asians					
	Not at all	28 (3.4%)	327 (39.4%)	423 (51%)	51 (6.2%)
	Several days	9 (4.8%)	71 (38.2%)	96 (51.6%)	10 (5.4%)
	More than half the days	2 (3.7%)	25 (46.3%)	25 (46.3%)	2 (3.7%)
	Nearly every day	0 (0%)	20 (45.5%)	21 (47.7%)	3 (6.8%)
Other races including multiracial					
	Not at all	6 (1.6%)	59 (15.5%)	246 (64.7%)	69 (18.2%)
	Several days	3 (4.1%)	13 (17.6%)	45 (60.8%)	13 (17.6%)
	More than half the days	0 (0%)	2 (16.7%)	10 (83.3%)	0 (0%)
	Nearly every day	0 (0%)	4 (25%)	9 (56.3%)	3 (18.8%)

## Discussion

Several studies have shown a positive correlation between BMI and depression-like symptoms [19,20]. In contrast to our initial hypothesis, when combining the prevalence percentage of being overweight or obese among men and women according to each depression symptom from the questionnaire, men who had a higher prevalence of being overweight or obese chose “more than half the days” or “nearly every day” for each depression category, except for the percentage of men who responded “more than half the days” when asked about being better off dead. In this study, 78% of the men who had trouble sleeping ”more than half the days” were categorized as being overweight or obese compared to 76.7% of women in the same categories. For individuals who had trouble sleeping “nearly every day,” overweight or obese men and women comprised 80% and 74.3% of those individuals, respectively. This differs from a study that reported a significant relationship between disturbed sleep and depression in elderly women, but not in men [21]. This also differed from a study that demonstrated poor sleep quality as being more prevalent among women compared to men, despite depression not being a significant contributor [22]. Our findings demonstrated a higher percentage of overweight or obese men compared to overweight or obese women who felt down or depressed “more than half the days” (79.3% vs. 75.3%) and ”nearly every day” (77.5% vs. 74.6%). This differed from a study that showed depression to be more prevalent in obese women compared to men [23]. Epidemiologically, depression and its symptoms are more prevalent in women than men, especially when we incorporate obesity [24]. Addressing depression in men is important, especially as we encounter cultural norms and attitudes that have associated a stigma towards seeking help for depression and other mental health issues [24].

Sleep problems were prevalent in the majority of overweight and obese men and women as well as all races [25,26]. One study showed that among patients with major depressive disorder, 84.7% also experienced insomnia [25]. Another study showed that patients with depression demonstrated characteristic electroencephalogram changes, including impaired sleep continuity, disinhibition of rapid eye movement (REM) sleep, shortened REM latency, and changes in non-REM sleep [26]. In addition, three possible mechanisms that link sleep and depression include inflammation, biochemical, and genetic mechanisms [27]. As it relates to inflammation, sleep deficiency contributes to increased levels of inflammatory cytokines. A strong relationship has been reported between inflammatory markers and depression. Biochemically, studies have shown a possible relationship between the dysregulation of catecholamines and neurotransmitters in REM sleep abnormalities. Genetically, the possibility of genes influencing insomnia and depression is apparent; however, more research is needed to further elucidate the relationship [27]. One study examining the relationship between sleep and depression in adolescents showed that those with depression experienced more wakefulness in bed, lighter sleep, and more sleep disturbance, but had little support for any predictive model showing depression to cause sleep disturbances [28]. With these findings in mind, the goal is to add to the fight against depression by examining the effects of obesity in different populations and their relationship with depression symptoms.

The BMI measurement used to screen and categorize the population into different weight categories is consistent with epidemiologic data, with the highest prevalence of obesity reported for non-Hispanic blacks, followed by Mexican Americans/Hispanics, non-Hispanic whites, and non-Hispanic Asians [9]. Although not statistically significant, each race had the highest prevalence of individuals experiencing depression-like symptoms (“more than half the days”) and (“nearly every day”) being categorized as overweight. There are a few exceptions to the above-mentioned finding. First, non-Hispanic Asians had the highest prevalence of those who had trouble concentrating “more than half the days” and “nearly every day” had normal weight. Second, non-Hispanic Asians who had the highest prevalence of those with poor appetites or were overeating “more than half the days” and “nearly every day” in a week also had normal weight.

The insignificance between all races for the majority of the symptoms implies an association between increased BMI and the prevalence of depression symptoms which is consistent among each race. This differs from a study that reported more of a variation between depression and obesity according to race and ethnic status [29]. There is a discrepancy in data between our study and the study by Bell et al. who included income as a variable that affects the incidence and prevalence of depression in the population [30]. Participants in the study by Bell et al., including white women, middle-income white men, and high-income African American men, demonstrated a positive association between obesity and depression, while there was no association between obesity and depression in African American women for all incomes. Another study focusing on racial differences among women demonstrated a positive association between obesity and depression in non-Hispanic white women, consistent with the findings of Bell et al. [31]. The researchers did not find an inverse relationship between obesity and depression in non-Hispanic black and Mexican American women. Meanwhile, Hawkins et al. evaluated racial, income, and age differences among male participants [32]. The researchers found that men were more likely to be obese or overweight and depressed if they were older, black, Hispanic, or were of low-income status. Future studies should focus on evaluating subtypes of black, Hispanic, and Asian communities in the United States to identify a higher risk of depression and obesity among these groups.

Another study evaluating the relationship between depression and obesity with diabetes as a co-variable found that factors associated with depression and obesity included being younger, female, having a lower education level, being functional impaired, and having diabetes [33]. The reverse causality between obesity and depression among these populations need to be further investigated.

## Conclusions

Our study demonstrated that obese men have a higher prevalence of depression or depression-related symptoms compared to women. The study also showed that overweight or obese men and women have a higher prevalence of depression/depression-related symptoms compared with normal-weight men and women. We identified a positive association between BMI and depression symptoms among each of the races studied. While no one race demonstrated a significantly better association between weight status and depression-related symptoms, it is crucial to identify those at risk for depression and screen for depression, despite any internal biases we may have. As such, universal screening for depression and obesity should be implemented.
